# Design and Evaluation of a Heterogeneous Lightweight Blockchain-Based Marketplace

**DOI:** 10.3390/s22031131

**Published:** 2022-02-02

**Authors:** Javier Antonio Guerra, Juan Ignacio Guerrero, Sebastián García, Samuel Domínguez-Cid, Diego Francisco Larios, Carlos León

**Affiliations:** 1 Department of Electronic Technology, EPS, Universidad de Sevilla, 41011 Sevilla, Spain; juaguealo@us.es (J.I.G.); sgarcia15@us.es (S.G.); sdcid@us.es (S.D.-C.); dlarios@us.es (D.F.L.); cleon@us.es (C.L.); 2 Department of Electronic Technology, ETSII, Universidad de Sevilla, 41012 Sevilla, Spain

**Keywords:** blockchain, marketplace, Internet of Things

## Abstract

The proposal of this paper is to introduce a low-level blockchain marketplace, which is a blockchain where participants could share its power generation and demand. To achieve this implementation in a secure way for each actor in the network, we proposed to deploy it over efficient and generic low-performance devices. Thus, they are installed as IoT devices, registering measurements each fifteen minutes, and also acting as blockchain nodes for the marketplace. Nevertheless, it is necessary that blockchain is lightweight, so it is implemented as a specific consensus protocol that allows each node to have enough time and computer requirements to act both as an IoT device and a blockchain node. This marketplace will be ruled by Smart Contracts deployed inside the blockchain. With them, it is possible to make registers for power generation and demand. This low-level marketplace could be connected to other services to execute matching algorithms from the data stored in the blockchain. Finally, a real test-bed implementation of the marketplace was tested, to confirm that it is technically feasible.

## 1. Introduction

The smart grid has become the solution used to integrate issues such as the appearance of renewable energy resources, changes in consumption patterns, the inclusion of electric vehicles, etc. In this sense, new technologies under the auspices of the Smart Grid concept have appeared to help manage power grids [1,2], with the management of the Distributed Energy Resources (DER) being a complex problem that presents important challenges [3].

In this scenario, it is important to consider that the installation of home photovoltaic (PV) systems has increased drastically in the last few years [4], mainly to reduce electricity bills, but also to reduce carbon footprints and to reduce dependency of the grid. In any case, these consumers could require electricity at some moments of the grid, but in other instances they could have a power excess, so they could feed this power surplus into the power grid, acting as producers. Therefore, these clients can be considered as DERs, following the prosumer paradigm [5]. All these actors make the smart grid difficult to manage, including precision aspects, such as grid synchronization [6] or prosumer energy matching [7].

Solutions to these problems present some scalability issues for managing power surplus management, especially with many unpredictable PV systems spread over the network [8]. Ideally, the systems need to keep the network stable. This implies trying to balance the surplus of power in the network, with its power demand using a matching algorithm. This could make other actors benefit and allow them to take advantage of this power surplus if they need it. Thus, both benefit from this power exchange.

The use of blockchain technology can be seen as a natural solution to overcome these problems [9,10,11,12,13]. Blockchain, by its definition, is a scalable, distributed, and decentralized ledger without the need for a central authority. Blockchain also allows the use of Smart Contracts (SCs) to execute algorithms within the network [14].

In this sense, this paper proposes a real implementation of a low-level power marketplace. This marketplace has been deployed using a blockchain over low-performance devices in the smart grid, which would even be installed inside the smart meters. This marketplace registers power production and demand on the blockchain through SCs. In this way, registered consumption and generation are stored in a public repository. Thus, the proposed marketplace could be applied to high-level or existing blockchains in established markets, and these main markets could be based on matching algorithm-based platforms or novelty markets based on blockchains, by means of the usage of sidechains. As described above, this blockchain is deployed over lightweight devices, which would be existing Internet of Things (IoT) devices used to process and generate data on the smart grid, allowing integration between these technologies.

However, to perform this, it is necessary to consider that the number of actors in the proposed blockchain could vary: prosumers, facilities, or external regulators, which could lead to the appearance of some problems in the blockchain network, such as synchronization faults or network overload [15]. In this paper, a proposal will be addressed that will be scalable, to avoid these risks.

A connection is proposed as close as possible between the smart meters of prosumers and the blockchain, even using the existing hardware inside the smart meter if possible. This approach involves taking the data closest to the data generation [16], ensure its veracity, and setting the data into the blockchain as quickly as possible, without the need for expensive computers or servers, which may have a high power consumption. However, it forces the use of low-performance economic low-performance devices on prosumer, aggregator, or retailer installations to maintain the feasible installation.

In this sense, this paper analyzes the application of a lightweight blockchain algorithm on low-performance devices for the design of a decentralized electrical marketplace, where prosumers and energy retailers can exchange directly and automatically among themselves. For that, it takes advantage of low-performance IoT nodes to act as blockchain nodes, by implementing a consensus protocol that does not require a high processing time or a graphic processing unit (GPU) to calculate complex and expensive calculus but is able to provide security and guarantee the quality and traceability of the data inside the blockchain. Furthermore, some SCs are implemented to automatize the power generated in the marketplace. All this architecture was deployed over real devices, tested, and compared over a similar blockchain network deployed on a personal computer.

The rest of this paper is organized as follows. First, Section 2 provides a brief overview of the integration of blockchain into IoT systems and some existing applications in the power sector. Later, Section 3 has an approach of different ways to implement the proposed solution. Then, the proposed architecture for the creation of the marketplace is shown in Section 4. After that, Section 5 shows the devices that are used in the designed marketplace. Then, how the marketplace is deployed in a real environment is presented in Section 6. Section 7 shows the obtained metrics and the comparison of these with a typical blockchain network. Finally, in Section 8 some conclusions are made, and possible future work derived from this paper are discussed.

## 2. Review of Blockchain Applied to Energy Markets

Energy retailers have been using blockchains more and more frequently in the last few years [17]. This technology allows them to save and trace electrical information about consumers [18]. Recently, the usual consumer profile has changed [19]. Many users install new renewable energy resources, such as PV panels, in their homes. Consumers who have installed resources such as these have seen their energy bills reduced, as part of this, demand for power is compensated by the generated one [20]. In addition, some of them now have no dependency on the power supplied, which means they are no longer actors on the power grid. Furthermore, in some cases, renewable energy resources produce so much power that is partially returned to the grid. In this scenario, the consumer has evolved into a generator and consumer, called a prosumer [21]. Therefore, the original purpose of the blockchain, which is to register only consumers in the smart grid, has become obsolete in many cases. The proposed marketplace overcomes this problem, allowing for generation and demand records.

Another challenge in DER management is the implementation of the electric vehicle, which could work in the local smart grid as a load or as a battery, known as a vehicle-to-grid paradigm [22,23]. This, as a new actor in the system or a new source for an existing user, requires new solutions, in which the blockchain could act as a guarantor of security or traceability, as [24] shows. The authors used blockchain technology to create a secure identity mechanism to implement a secure charging system. In the proposed marketplace, the electric vehicle could be part of any actor on the blockchain, and there would be no difference in the behavior of the network.

The integration of blockchains into power grids is not a new challenge. It is widespread in the literature, where many challenges have been exposed. Ref. [25] exposes some of the challenges blockchain needs to overcome in the power sector and briefly shows the advantages of using it on IoT platforms. That work also mentioned the Brookling Microgrid [26], one of the first studies of a real microgrid, which demonstrates that blockchain is a suitable technology to provide microgrid marketplaces. They proposed a market divided into seven components, using blockchain as an information system in the most specific component of the market. In [15], the authors expose some use cases for the implementation of blockchain networks in smart grids, such as peer-to-peer, power trading, and power distribution. Throughout this paper, it is shown how most of the challenges have been overcome, such as the increase of disk size among the blockchains, or the use of heavy consensus algorithms, which will be discussed and partly solved in this paper.

There have been some attempts to merge IoT and blockchain with different approaches. Some research has been conducted on the hybridization of both technologies [27]. In this sense, the blockchain–Internet of Things paradigm has been analyzed, such as in [28], to reach trends in industry and academia, using innovative review methods. The authors in [29] go a step further and introduce 5G networks to the scope of the IoT to study their convergence with Blockchain. Regarding the practical applications for both technologies together, in [30] the authors provide the execution of SCs for access control on IoT devices, using blockchain technology to guarantee secure accesses. However, the blockchain network is implemented over servers, using a heavy consensus protocol. The authors of [31] use Raspberry Pi as an example of an IoT device for a scalable Blockchain-into-IoT scenario for secure information sharing. Despite this, it continues to use non-IoT devices as nodes of the blockchain, leaving Raspberry Pi as an IoT-device server. More similar to the approach of this paper is the use of Raspberry Pi as blockchain nodes proposed in [32], with devices connected to a blockchain network. Unfortunately, the use of IoT devices such as these has no more use than as nodes in the network, wasting its potential to work in other applications, as proposed in Section 1.

A close implementation of IoT nodes on blockchain is [33], where the authors proposed a framework for distributed applications in an IoT network with blockchain. That solution introduces another layer to its architecture, with the use of a specific server to deploy the framework. Multi-layer architecture was also needed for the investigation in [34], although it is deployed over a Field Programmable Gate Array (FPGA) device and does not provide a lightweight protocol to the blockchain network. In this paper, it is explained that there is no need for servers or complex devices to deploy a blockchain for the use of IoT at the same time. The same conclusion is reached in [35]. In that, a power trade system using SCs, using Raspberry Pi as nodes for their test network is proposed. Although they mention the use of whisper as a messaging service that the blockchain provides, it is not very clear which algorithm they used to reach consensus in their network, as they talk of Proof-of-Work and mining blocks. This made the nodes unusable for other purposes, more than supporting the blockchain.

## 3. Advantages of the Use of a Blockchain-Based IoT Marketplace

**Figure 1:**
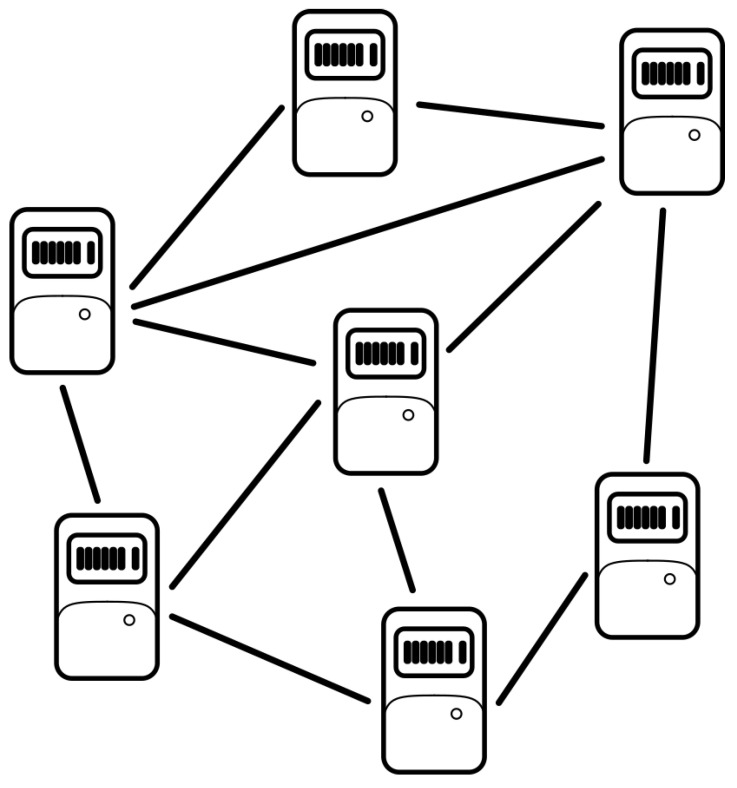
Nodes in a decentralized network.

As mentioned above, the main technology on which this whole proposal is based is blockchain, which is defined as a distributed ledger technology. In blockchain, as in traditional ledgers, all information recorded can be considered true and immutable. The blockchain is composed of blocks, which are a set of transactions with some metadata information (i.e., the information of each transaction codified through hash functions) to prevent public access to all data and guaranteed anonymity. The main properties of a blockchain are as follows:

- Decentralization
  Blockchain was born as a decentralized technology to avoid any unique authority in the network. Instead, it is distributed into different nodes, with multiple connections among them, as shown in Figure 1. This creates a solid and reliable network with many benefits, such as efficiency and data propagation. This implies that if a node is down for some reason, the rest of the marketplace will continue working without fault.
- Immutability
  The moment that a block is validated by the network and linked to the chain, this information is very difficult to modify; the difficulty of doing so increases with each new block added to the network. Therefore, it is very difficult to modify a validated block in a chain. With application to the marketplace, it is guaranteed that the power data in the blockchain will not be modified by a third party unless that party manages to gain control of almost all nodes.
- Distributed consensus
  It is only possible to add a new block to the blockchain if most of the nodes agree on adding it. This is called validation of a block. Thus, a consensus is needed between the nodes of the network. This consensus means that the responsibility is decentralized, as there is no main individual responsible for a block’s creation. Decisions in the blockchain network are only made by a consensus in the network. This reduces possible bottlenecks due to the dependence of a unique validator. The consensus also helps to increase network reliability because, as mentioned earlier, it would be necessary to attack all validators to modify the data in the blockchain. This implies that the consensus of the network is distributed, and the truth of the information is the same for the network. Thus, if a malicious node in the marketplace changes its own information and tries to share it with the rest of the network, it will be discharged, and the misinformation will have not be distributed to the network.
- Traceability
  The information in all of the nodes of the blockchain is public. Therefore, it is possible to access any transaction recorded in a block and obtain its hashed information, such as the user who performed the transaction, the user to whom it was sent, or the timestamp of it. Thus, if a user knows the hash of some data, it is possible to obtain all transactions related to these data and the block where they are stored. This ensures the traceability of any data in the network, so it is possible to trace the power data of a user in the marketplace.
- Speed of transactions
  Integration of the IoT infrastructure on the same nodes as the blockchain network allows the possibility of communication between them. This communication could be performed using less information than in a conventional blockchain, which increases the speed of transactions and, at the same time, the time needed to create a block.
- Reduced cost
  Although it is not relevant from a technological point of view, it is true that integrating IoT and blockchain in the same device would result in the use of fewer devices than using both technologies separately in the same scenario. Therefore, fewer devices implies fewer failure options and less maintenance. This is directly related to a decrease in the cost of implementing the technology.

The number of different blockchain networks and applications referring to the access to these networks is counted by dozens [36,37]. Therefore, it is quite a difficult decision about the optimal blockchain network that meets the objective of the marketplace proposed by this paper, as is a blockchain that runs for short periods of time, allowing the device to be used also as an IoT node.

In this sense, several consensus algorithms have been evaluated, with its main characteristics in the following subsection.

### Consensus Algorithm

A consensus algorithm is named as the algorithm required to validate the data contained in a block by all nodes before adding to the tail of the blockchain. By this, it is guaranteed that the data inside the block have been defined as true by the majority of nodes in the network, which means that they have no false or manipulated data before introducing them into the blockchain. Due to that, consensus algorithms have to be complex, involving a large percentage of nodes in the network, whether creating the proposed block or validating it. These consensus algorithms have already been studied, obtaining properties such as the number of rogue nodes to fail [38].

As the blockchain network has nodes, such as low-performance devices, it is necessary that the consensus algorithm is secure, fast, and lightweight. Some of the most extended consensus algorithms and their suitability for the proposed architecture are presented below. Although normally blockchain networks have their own consensus protocols, most of them are based on these consensus algorithms.

- Proof of Work
  The first consensus algorithm proposed for the blockchain was Proof-of-Work (PoW), defined for the Bitcoin blockchain. PoW based its effectiveness on using all available computational resources to solve a cryptography problem to create a valid block in the network. The solution to this problem consists of finding a nonce, a number that makes the hash function of the whole block match any condition, which differs according to the difficulty of the network at that moment. Once a block is created, the rest of the network is responsible for validating it. Although it is very costly to create a block, it is quite simple to check if it is valid or not, due to the use of hash functions as part of the cryptography problem. With *N* denoting the nonce, $P_{hash}$ the hash of the previous block, $T_{x}$ all the transactions inside the block to be created, and Target the difficulty of the network, the PoW requirement in Bitcoin is defined as the following equation:
  (1) $$H\;(\;N\;||\;P_{hash}\;||\;T_{x}\;||\;T_{x}\;||\;...T_{x}\;)<Target$$
  The above equation was obtained from [39].
  Due to the fact that PoW needs to use the most resources of any algorithm, any node has to find the nonce to create a block [40], this consensus algorithm is discharged in the marketplace.
- Proof of Stake
  The Proof-of-Stake (PoS) consensus algorithm is based on the following premise: nodes that have the most coins in a blockchain network have the least interest in attacking the network [41]. On the basis of this, PoS does not need to solve any complex problem to create a block. Instead, PoS bases block creation on a stake of coins for each node in the network. Thus, the node that stakes the largest amount of coins is declared the creator of the block and is in charge of adding the data to the new block and linking it to the main blockchain.
  Besides all its advantages, PoS has a main problem. As the consensus in this algorithm is reached by staking coins, it would be possible that some nodes join themselves and transfer funds between them to have enough coins to win any stake in the network. Due to that and some other related problems [42], PoS is not a suitable consensus algorithm for the proposed marketplace.
- Proof of Authority
  Another alternative to reach consensus between nodes is the Proof-of-Authority (PoA) consensus algorithm. In PoA, the main difference from other consensus algorithms is that the trust of the blockchain network is based on identity and reputation [43] and does not requires any currency exchange. Nodes of the network need to be firstly authorized as voting nodes to avoid new nodes tampering the next steps of the algorithm. Then, the voting nodes decided, based on the reputation of the nodes, which nodes are voting to be selected as validator nodes. The selected validator nodes finally need to show themselves to the network, in return for being able to add new blocks to the blockchain. In this consensus algorithm, as for the PoS consensus algorithm, a block is said to be signed instead of mined, and it does not require a high computational capability per GPU. In case the remaining nodes detect a rogue block, incorporated by a malicious node, the node will be identified and removed from the network. The main disadvantage of the PoA algorithm is that if an authority could have control of half plus one node of the network, the consensus algorithm will be tampered with, similar to PoW. Therefore, increasing the number of nodes in the network (i.e., with more prosumers in the proposed market), the security of the system increases.
- Practical Byzantine Fault Tolerance
  The practical Byzantine Fault Tolerance (pBFT) is an algorithm that ensures the unalterability of the network messages. Its consensus is based on state machines’ replication, i.e., the consensus is reached when two thirds plus one of the nodes answers the same state for the request sent by a client, which is another node. So, it is possible to support rogue nodes if they are fewer than a third of the total nodes in the blockchain network. As any client needs to communicate to all nodes in the network in each request, this consensus algorithm generates excessive network traffic, which is against the purpose of creating a lightweight blockchain proposed in this paper.
- Proof of Elapsed Time
  The Proof of Elapsed Time (PoET) is based on random time windows, in which a participant of the consensus competes to generate a block, develop a cryptographic proof, and send it to a controller. The controller verifies the proof and accepts the block to proceed. The controller then creates a new time window, and the participants compete again. Although the consensus algorithm is free to use, it is developed using technology introduced by Intel hardware. Due to this, it is not suitable for use in the proposed lightweight devices.
- Proof of Importance
  The Proof of Important (PoI) consensus algorithm seems like the PoS algorithm, with a main difference: the selection of the node is made by the score any node has. This score is related to the quality of the node and refers to the number of recent transactions, the vest of currency for the creation of new blocks, the network traffic, and more. Due to the fact that the more network traffic a node has, the better score it is able to reach, this consensus algorithm favors this feature, which is against the proposed lightweight blockchain.

As the blockchain network has nodes, such as low-performance devices, it is necessary that the consensus algorithm be secure, fast, and lightweight. There are other consensus algorithms based on disk usage, specific hardware chips, or other properties. Due to the hardware and software restrictions of the devices used for implementation, all of these alternatives have been discharged.

Finally, the consensus algorithm for the marketplace is PoA. It does not require many processing resources and could act during short periods of time, leaving the rest of the time for other purposes of the device. As will be seen later, PoA has solid, reliable, and supported alternatives in the proposed devices, which reinforces the decision about which consensus algorithm to use.

## 4. Proposed Architecture of the Distributed Marketplace

**Figure 2:**
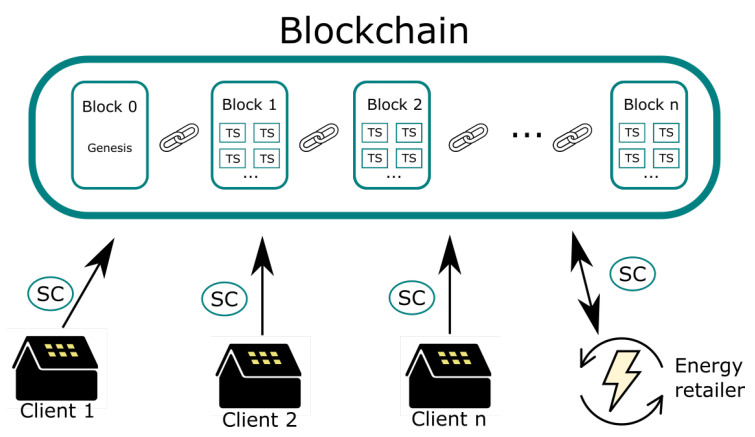
Marketplace scheme.

The general architecture of the proposed marketplace is depicted in Figure 2. In this figure, prosumers and energy retailers have access to the blockchain marketplace, in which resources are available to the participating actors, either in terms of generation or power offers. The registers of this generation or power offers are stored at the source, establishing a high grade of quality and traceability of energy in a permissioned ledger (only the clients with the device could access to the platform). Thus, this is very useful for a marginal power market, providing an additional and decentralized veracity.

The definitions of the participants in the marketplace are as follows:

- Client or prosumer: as described above, they should act as a consumer or a generator. In both cases, the proposed node will be deployed on the smart meter (or be the smart meter by itself) to obtain the energy data each fifteen minutes, in the best case. This node will send the data to the energy retailer database and trigger the SC to publish both powers: surplus or demand.
- Energy retailer: It is responsible for the installation of the nodes and the connection between them. It may have a register of all information registers in the blockchain network within its database.

The data flow of the information in the marketplace results as follows. Inside the client supply, there is one of the proposed devices, working as an IoT and a blockchain node.

1. The IoT node behavior registers any power data, sent each fifteen minutes periodically. This information includes the power surplus of the prosumer. This will be a positive number, in the case of power generation, or a negative for power demand. In the remaining time, all nodes work as blockchain nodes, reaching a consensus of the blocks in the network using the PoA consensus algorithm [44]. These blocks contain transactions that consist of hashed data and SC information.
2. At the moment a surplus of power data is received, the node checks through a SC if the user is registered into the blockchain to send data. If not, an SC is called to register the user on the blockchain. If the user exists in the blockchain network, the blockchain node will send the power surplus data inside a generation SC or a demand SC, depending on the sign of the power surplus data. The data are also sent to the energy retailer to store them in its database, before the new data is registered. It is important to note that the marketplace does not perform any data processing, only stores the data sent by the SCs, which consist of hashed data and not the data itself.
3. The high-level or primary blockchain periodically executes (each 15 min or each time required by the retailer) a matching algorithm. It matches the generation registers with demand registers in the blockchain, checking the source of generation and offers.

As can be seen, the blockchain is a crucial part of the marketplace, as it is the basis of the traceability of the entire marketplace and needed in the matching process that can act with the independence of the retailer.

In this sense, it is important to find a valid architecture for the proposed marketplace, searching for a trade-off between computational needs, consumption, and cost. To reach the correct implementation of the blockchain marketplace, five main architectures have been analyzed:

1. Scalable sidechain
  In this case, it would be proposed to have only one sidechain in the whole marketplace. A sidechain is defined as a parallel blockchain, which works while the original blockchain properties are locked so that the common data between those two blockchains have no potential for conflict. This sidechain has its main advantage in its scalability property, which allows it to reach all the actors in the energy retailer. Unfortunately, it would require developing a specific sidechain core, compliant with ARM architectures, this is required, as ARM is currently state-of-the-art among computational capabilities and consumption. Although it has been studied, this restriction provides a non-valid solution for this marketplace.
2. Using Hyperledger-based distributed ledger technology.
  This option requires distributed ledger technology (DLT) that uses some of the solutions provided by the Hyperledger blockchain [45]. Some Hyperledger applications do not have a non-native ARM implementation, such as Hyperledger Besu [46], so it is not known if it would be possible to adapt it to the requirements of this paper.
3. Using alternatives based on consensus algorithms by capacity, space, or time.
  This kind of consensus algorithm seems to be appropriate for this project, as it reduces computing and consumption of the nodes. Due to the limited storage that nodes have, it would be difficult to implement this alternative with effectiveness. Therefore, it is discharged.
4. Using IoT-oriented alternatives
  There are alternatives that are not blockchains, but would be considered blockchains or DLTs, such as IOTA [47]. It has been tested in a private network (Tangle) over a virtual machine in a PC, which allows the installation of a Hornet service in lightweight nodes. However, after that, some shortcomings merged in terms of compatibility and interoperability. Although it appears that this problem will be solved in the next version of the client, right now it is not a real solution for the purposes of this paper.
5. Using Ethereum-based solutions
  There are some Ethereum-based blockchains which are able to be used in ARM architectures and also different options related to Ethereum that could be used. Moreover, the main structure for this type of network eases the interoperability and the connection with other sidechains if necessary. In fact, commercial solutions exist for this purpose. Therefore, it is the most viable alternative for this paper, and the one we finally used.

Once it is decided to deploy a blockchain based on Ethereum, there are two main options available to be implemented on the proposed devices. Both options involve using a lightweight client to deploy the blockchain written in the Go programming language. Therefore, a decision is needed between an Ethereum-based blockchain, such as Quorum, or the Ethereum client, with its implementations called GoQuorum and Go Ethereum. Both alternatives have the option of using a consensus protocol based on Proof-of-Authority, called raft and clique, respectively. The decision to use one of them is based on its maturity state, where Go Ethereum is older and more developed than Go Quorum. Therefore, Go Ethereum is the chosen implementation for the marketplace. In all nodes, a standalone Go Ethereum client, called Geth, has been installed. Geth provides enough tools to develop a full Ethereum node with the desired consensus protocol and the deployment of SCs on different nodes.

Geth only allows the execution of the blockchain on the CPU, as it is not guaranteed that all devices could have a dedicated GPU for this purpose. Moreover, it is not required for a PoA. The chosen consensus mechanism would also have a low impact in the case of GPU processing, as clique has been designed to be lightweight, in contrast to protocols based on PoW.

## 5. Energy Market Testbed Description

As described before, to deploy the blockchain network, we decided to use low performance devices, which act as an IoT node and a blockchain node at the same time, without loss of performance. Although many devices exist which have a special purpose for IoT, we decided to use some generic and popular devices that have a reasonable price. This is a frequent requirement, especially for large deployments, as the proposed marketplace can affect production. These devices have been implemented on their own smart meters, or very close to them, in several projects [48,49,50].

The three devices used as nodes for the marketplace are as follows:

- Raspberry Pi 3 B+ (RPi3), a low-cost single-board computer. Its central processing unit (CPU) has been developed over an ARM architecture and has 1 GB of random access memory (RAM). Its disk size depends on the size of the memory card inserted into it. Its consumption is low, and it is commonly used in several applications, such as edge computing in an IoT context [51] or sensor network management [52].
- Raspberry Pi 4 (RPi4) is an upgraded and more recent version of RPi3. The main differences are that RPi4 has better CPU performance and different versions with different sizes (RAM), in exchange for slightly higher consumption.
- Jetson Nano (JNano) is an embedded computing board with a target-embedded IoT application and robotics [53]. Its CPU is also ARM-based, the same as the other devices, but JNano has some extra hardware features, such as a GPU. It has more power consumption than the other devices but consumes power without excess.

Table 1 shows an overview of the relevant specifications for the three devices used for this paper.

All three devices used in this paper have an ARM architecture. There are many blockchain applications that have been deployed only for x86 or x86_64 architectures, so it would be very difficult to run this network inside any of the devices mentioned above. Fortunately, there are also some kinds of blockchain that have a native implementation for ARM architectures [55]. That implementation is highly different from the one used in servers or specific devices for mining or signing blocks, such as application-specific integrated circuit (ASIC) devices [56].

Apart from that, the existence of an ARM-architecture client is not enough to ensure the blockchain will work properly. Both RPi3 and RPi4 are built on an ARMv7 architecture, whereas JNano has an ARMv8 architecture. Although this may be seen as a small difference, it would make any blockchain client suitable for RPi3 or Rpi4 and not fully compatible with JNano. Sometimes, it is possible to avoid this restriction from building from the source. However, other times, even building from its source code does not guarantee that the blockchain runs properly. Therefore, to meet the heterogeneous blockchain network, some blockchain clients are discharged due to this restriction.

These devices are used for validation purposes. Their main goal is to test the suitability of developing a blockchain for a marketplace with low-cost, low-power consumption and limited computational capability hardware. Commercially, the marketplace deployment can be performed on ad hoc devices with processors based on the tested ones, or similar, as a function of their production costs.

In this sense, it is important to note that it would be possible to deploy the blockchain marketplace using the same device for all nodes. However, this paper tries to show that the proposed solution is heterogeneous, without dependence on a single commercial solution. If the proposed marketplace can be fully executed on a device with lower computational requirements, it can be executed on the devices of other manufacturers if they have equal or superior computational capabilities.

## 6. Marketplace Testbed and Results

The deployment of the experimental marketplace testbed is divided into three parts. First, the optimal deployment of the architecture to perform the blockchain marketplace and its nodes is shown. Then, it is defined as the genesis block of the blockchain that allows the network to be lightweight, fast, and secure. Finally, the Smart Contract (SC) used in the marketplace is commented on.

**Figure 3:**
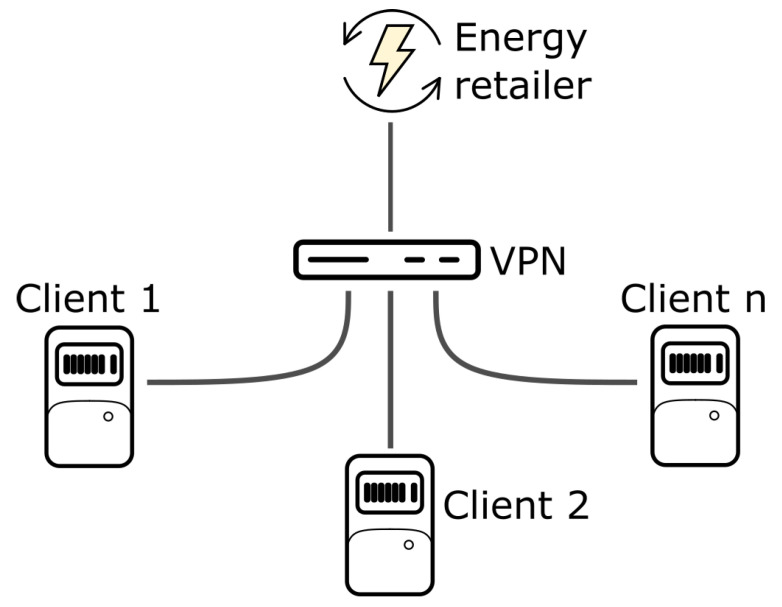
Proposed network scheme.

The proposed network is shown in Figure 3. As can be seen, clients (i.e., the prosumers) have their nodes connected to the smart meter. To guarantee security and avoid other risks derived from the use of public networks for the communication between nodes, it is a necessary, efficient, private, and alternative connection. Due to that, all these nodes exist in a virtual private network (VPN), enabling the required exchange of information in the blockchain and being the retailer responsible to maintain this network. Each client must have an internet connection, with different providers and different internet restrictions, such as firewalls or Carrier Grade Network Address Translation (CG-NAT). However, for the execution of the selected consensus algorithms, the clients must have a direct connection among them, to exchange information. In this sense, more complex communication protocols can be implemented, but VPN solves these problems in a natural way, allowing direct communication.

**Figure 4:**
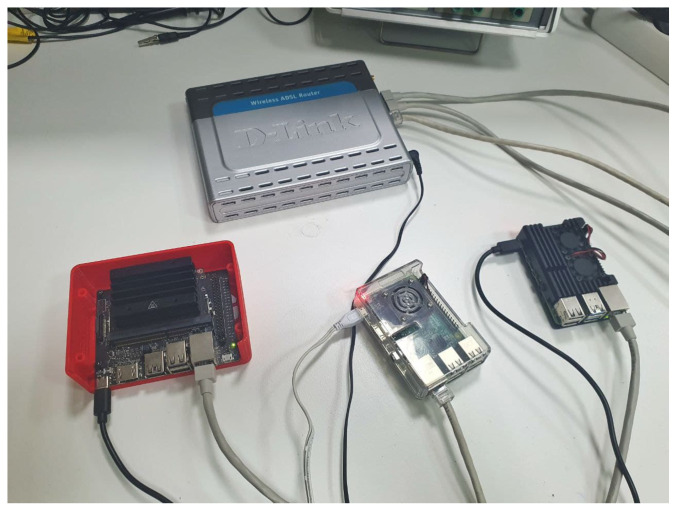
Marketplace-tested devices: **up**: switch; **bottom-left**: JNano; **bottom-middle**: RPi3; **bottom-right**: RPi4.

In the case of the testbed, this structure is simulated with three nodes connected in a private network. As described above, these nodes are based on low-performance devices. Thus, the network is finally as follows in Figure 4, where there are three nodes in the network, composed of a JNano, a RPi3, and a RPi4.

These three devices hold the following software:

- Raspberry Pi 3 and 4
  Both RPi3 and RPi4 contain analogue software. Both are built on an Ubuntu Server operating system (OS). Ubuntu is a general-purpose Linux-based OS, which has versions for many different architectures, among them ARM architectures such as those deployed on these devices. A server version of the OS has been installed to save as many computing resources as possible, avoiding any graphical interface.
  After that, Geth was installed on both devices. This was conducted by downloading ARM-specific compilations from the source page, after downloading GoLang packages for ARM, implemented in the native package download application for Ubuntu.
- Jetson Nano
  JNano software is built over an OS as part of the Jetson Nano Developer Kit. This is a set of tools that enables the OS to work properly on a JNano. It is a Linux-based distribution, built specifically for this device, with many similarities with other operating systems such as Ubuntu. GoLang was then installed and Geth after that, as Raspberry Pi 3 and 4 had previously been installed. In this case, Geth has a native download from the OS package manager.

Next, the necessary configurations were made to run the blockchain network. These steps are all the same for the three devices. As the network has been disconnected from the internet, it is necessary to manually set the date and hour for every system. This is performed using the *date* command. It is important to set the hour exactly: a difference of synchronization of twelve seconds is enough to avoid the nodes from connecting to each other. After that, the genesis block is generated and used to start the blockchain, where all nodes share the same network identification; the genesis block has the same Geth configuration parameters to ensure communication between nodes. Finally, the connection between all of the nodes is established, and then starts the process of validating and signing blocks.

Initially, all nodes were set as full nodes. This means that each node in the blockchain network has a complete copy of the chain. This can easily be changed in case some problems arise related to the available space on disk. In that case, the node would be set as a light node, which contains the latest blocks of the blockchain, saving disk space.

### 6.1. Genesis Block

The genesis block is the zero-block of any blockchain. It is the only block in every blockchain that has no reference to the hash of the previous block. Due to that, the genesis block has specific content. It is in charge of defining some parameters of the blockchain network from the point after its creation, and it is impossible to modify them once the network has started, similar to the content of any other block in a blockchain.

The genesis block is defined in Geth with a Javascript Object Notation (JSON) file called genesis.json. It has many parameters used to configure the entire blockchain network, such as chain ID, the type of block used, use of any hard fork in the network, or the consensus protocol to be implemented, among others. All these specifications are in accordance with Ethereum Improvement Proposals (EIP) [57].

According to the objectives of this paper, the genesis block must be the lightest possible. This is due to the fact that it is needed to avoid all unnecessary configurations, to reduce communication between nodes as much as possible and to configure the consensus protocol to be the one that consumes fewer computational resources of the devices. The genesis block is also responsible for other parameters, such as the gas limit for transactions, the first account of the network, which correlates with the first signer node of the network, and the initial accounts and initial amount of currency for each account.

### 6.2. Smart Contracts

Smart Contracts are pieces of runnable code inside the blockchain [58]. With SCs, any user of the blockchain could execute a computer program inside the blockchain network. Thus, the applications of the blockchain increase. Before SCs, any blockchain could only store transactions inside the blockchain network. Now, SCs allows any user to send a transaction to the account of an SC, which triggers the execution of the code to perform some operations. As the blockchain network is decentralized, any node in the blockchain network could write and store an SC in a block. After that, the SC must be deployed to be used by a node of the network. Once the SC is deployed, the address of the SC is public, and any node of the blockchain could access it. Fortunately, the SC could have some restrictions in its code, so that no node would be allowed to execute it. The SC source code is not automatically public to the network, so it is possible to keep the source code of the SC private, with access to the SC source code only available for the node that creates it.

For Ethereum-based blockchains such as Geth, SCs are written in a programming language called Solidity. As these types of blockchain network are Turing-complete, the way an SC is programmed is quite similar conceptually to any other code in high-level programming languages. It has some limitations due to restrictions of the blockchain and the way it is managed, such as loops in code or handling data outside of the blockchain.

For this project, a SC is proposed as the best way to deploy a marketplace inside the blockchain. However, SCs are not the only alternative to deploy a marketplace. Alternatives, such as the use of oracles [59] to create a marketplace outside the blockchain and then introduce the data into the network, could be also available. This option has been discarded for various reasons:

- It is not on approach to storing the data closest to the place where it was generated or obtained.
- It is possible to tamper with the data, so that, in the blockchain, different data could be stored than in the primitive one.
- It requires more complexity to afford it, as it implies adding an oracle between the data and the blockchain network.

For the proposed marketplace, two main SCs have been deployed along with an auxiliary one:

- Register
  This contract allows the registration of the actors involved in the marketplace. Thus, it is possible to register a prosumer, identified by its Universal Supply Point Code (USPC), or a retailer, identified by its VAT number. It has enabled some restrictions, such as the prohibition of an external actor from participating in the marketplace, as will be discussed in the following section. It also has some extra functions to retrieve some information in the contract, such as the number of actors in the marketplace, or the count of the prosumers.
- Operation
  The second SC has the register of any operation inside the marketplace. Therefore, in the blockchain, there will be information about any generation and offer that occurs in the marketplace. The producer of this generation, the price of this generation, and the generated power are all stored in the generation register. In the case of an offer register, the blockchain stores the information of the producer and, as in the case of generation, the price and the amount of power. The SC is in charge of storing this information in the blockchain with a corresponding timestamp.
- Auxiliary contracts
  For the proper functioning of the SCs above mentioned, it is also necessary to create some more auxiliary contracts, which ensure the register and operation contract will work as desired. Therefore, the option to delete a registered actor in the system was developed, but only restricted to its own actor. For this, it was necessary to create other contracts that ensure the owner of the contract. Finally, an auxiliary contract was created to ensure that any actor in the marketplace had enough funds to execute it.

**Figure 5:**
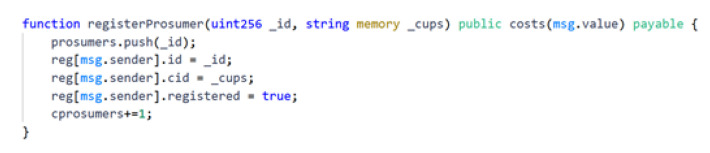
Example of the *registerProsumer* function inside one of the Smart Contracts.

As described above, all these SCs have been written using Solidity, and it is fully compatible with the Ethereum blockchain and its derivatives. This was a possibility because of the Ethereum Virtual Machine, the environment where all Ethereum-based blockchains work. This allows the execution of the SCs in the same blockchain where the blocks are signed. This implies that the Geth compilation that is running on the nodes has native compatibility with the SCs. An example of the code of a SC function is shown in Figure 5.

## 7. Performance Results Obtained

As described above, the proposed blockchain marketplace has been deployed on a testbed. That is, the blockchain has been executed from scratch on three nodes on these devices: a RPi4, a RPi3, and a JNano. According to the specifications of the device, the genesis block was designed with the following roles: the RPi4 will act as a validator node and as a signer node, as it is slightly more powerful in terms of CPU processing. Therefore, RPi3 and JNano will act as regular nodes on the blockchain. This is a sample of the proposed blockchain, where a few nodes would act as signers, based on their computing capacity and network connections.

The blockchain was left running for several days, while simulating the marketplace. i.e., the simulation of transactions required each 15 min per actor. After that, some key metrics were acquired, which have particular relevance to demonstrate some properties of the blockchain marketplace. These metrics have been acquired for the three nodes and compared between them. To avoid problems related to the specific time of data collection, relative metrics have been acquired, whether as the hourly average of data or in percentages.

The description of the metrics in Table 2 is as follows:

- Disk_ReadDatarefers to the amount of data read on the disk in bytes.
  The data read from the disk are used to evaluate the performance of the node. It is desirable that the node should read the least data possible, to avoid processing time wasted in reading data operations.
- Disk_WriteData describes the amount of data written onto the disk in bytes.
  This metric is directly related to the necessary disk size in case the node was a full node of the blockchain
- Network_InboundTraffic indicates the inbound traffic in the p2p network in bytes.
- Network_OutboundTraffic indicates the outbound traffic in the p2p network in bytes.
  These two metrics are relevant in the case of a network failure. The more traffic inside or outside the node, the more synchronization problems that would exist. It also refers to the network speed needed to run the blockchain successfully.
- Memory_Allocated refers to the amount of memory assigned to the blockchain in bytes.
- Memory_AllocatedTotal indicates the Memory_Allocated metric related to the total RAM each device has.
- CPU_ProcessesTime describes the total time the CPU has taken during the blockchain processes.
  Finally, these two metrics are crucial to evaluate whether the node could be used for other tasks at the same time as running the blockchain network.

The results of these metrics obtained at all three nodes are shown in Table 2.

It is possible to obtain some conclusions by referring to these data:

- The blockchain network is technically feasible. It has been deployed, started, and run for hours without any error or strange behavior.
- Each device has different performance. In fact, the device with lower specifications in terms of CPU speed and RAM generates modest metrics compared to other devices. However, it is more than enough to run the proposed blockchain network.
- There is virtually no limit on disk usage. If we take the worst case, which is 11.5 KB per hour for RPi4, it would take roughly 10 years to occupy 1 GB of disk space.
- The blockchain does not need much network speed. A simple General Packet Radio Service (GPRS) connection has enough speed to obtain a successful connection between nodes.
- Nodes may have enough features to run other tasks while the blockchain is running. Over 90% of free CPU time in any node, and less than 1% of RAM allocated by the blockchain, make these low-performance nodes suitable for executing some data acquisition tasks. Thus, it is possible to obtain data and upload them to the blockchain from the same device, without any other connection or device that could manipulate the data quality.

To contrast the results given by the marketplace, it will be compared with a blockchain with similar characteristics, deployed over a PC instead. The PC has an Intel Core i7-10510U @1.80 GHz CPU with a x86_64 architecture and 8 GB RAM, running Ubuntu Server OS. Specifically, it was deployed on three Geth nodes with a Docker container each. It has used the same genesis block and deployed the same SCs, and started and simulated the same transactions as the marketplace. The definition of the same genesis block implies that it uses the same clique PoA-based consensus protocol. Finally, the metrics have been obtained in the same way as the proposed marketplace.

**Figure 6:**
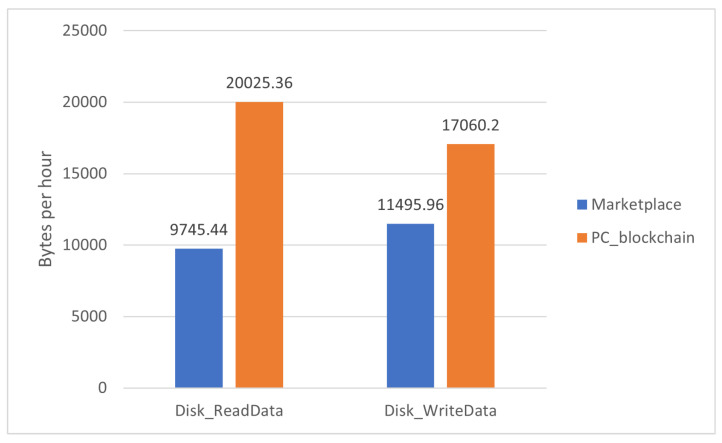
Comparison of disk usage metric between the proposed marketplace and an analogue blockchain over a PC.

**Figure 7:**
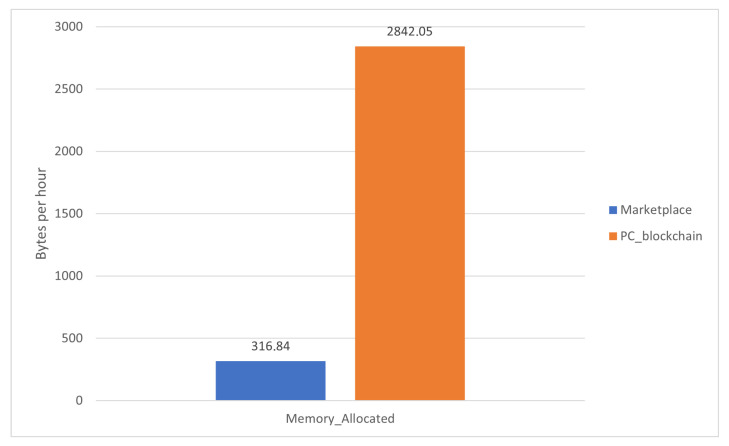
Comparison of the memory allocation metric between the proposed marketplace and an analogue blockchain over a PC.

**Figure 8:**
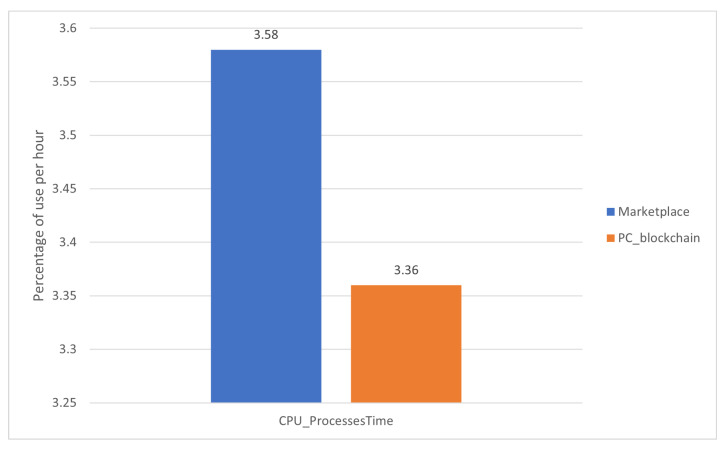
Comparison of CPU usage metric between the proposed marketplace and an analogue blockchain over a PC.

The comparison has been made using the worst node for each case. Those are:

- The most data read and written in Figure 6 corresponds to RPi4.
- The most memory allocated refers to the total available in Figure 7 corresponds to RPi3.
- The highest CPU percentage in Figure 8 corresponds to RPi3.

The proposed blockchain marketplace was labeled as Marketplace and the deployed blockchain nodes over a PC as PC_blockchain.

From these results, it can be determined that powerful devices will perform better in the proposed blockchain, as the three graphs show better results for the PC_blockchain alternative. Despite this, the marketplace proposed in this paper has enough performance to run the blockchain under sufficient conditions to work as IoT nodes at the same time.

To evaluate the scalability in the market, some benchmarks were performed on the weaker performing device, i.e., the RPi3, using the open-source benchmarking tool Hyperledger Caliper [60]. The following benchmarks were executed using the Operation SC:

- 1000 operations at 25 transactions per second (TPS).
- 1000 operations at 50 TPS.
- 1000 operations at 100 TPS.

We obtained the results shown in the next two tables with all transactions sent successfully in each round of the benchmark. Table 3 shows the send rate and latency of the transactions, while Table 4 focuses on the node throughput and the CPU in terms of average (avg) and peak use.

**Figure 9:**
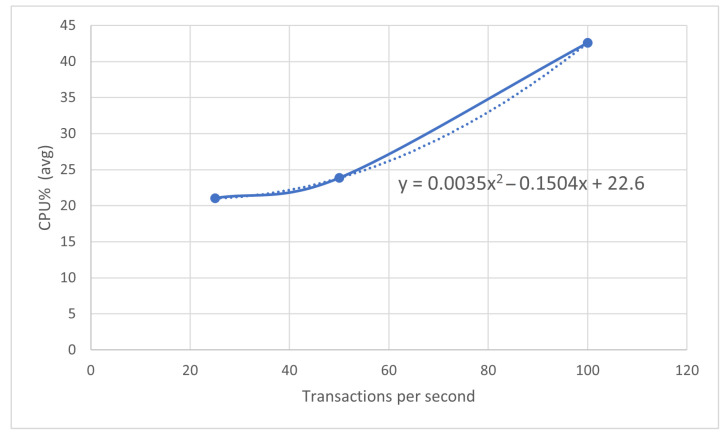
Percentage of CPU used for RPi3 and number of transactions per second.

To reach that number of nodes, the RPi3 could support sending transactions to the blockchain, and the average CPU % used is inferred, as shown in Figure 9.

Thus, if each node in the blockchain marketplace sends only one transaction per second, it is possible to reach over 128 TPS before the RPi3 reaches 100% of the CPU usage time, which means it is possible to support 128 nodes in the blockchain. Moreover, the data were registered every 15 min, so the number of real transactions was at least a third of the number of calculated transactions, and some time was needed for processing. Therefore, it is possible to say that the blockchain would support approximately 200 nodes without blockchain congestion, using only a device with lower CPU performance. Moreover, if a sidechain reaches its limit, a new sidechain can be added. This is why the proposed architecture can be used with an arbitrary number of prosumers.

**Figure 10:**
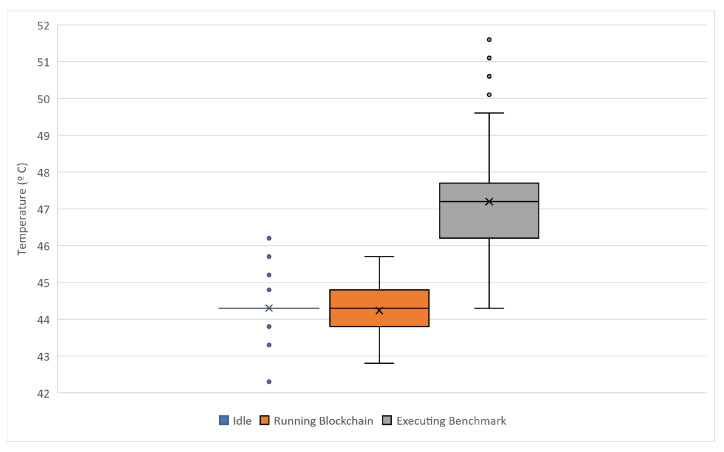
RPi3 CPU temperatures in different scenarios.

With respect to CPU usage in these compact devices, it is necessary to consider if the device would suffer from high-temperature issues. These devices are known to work at high temperatures, and high CPU usage would cause temporary or permanent undesirable effects, such as throttling [61]. Due to that, CPU temperatures were monitored every half second in order to control any possible risk if the device reaches high CPU temperature. This temperature, as shown in Figure 10 has been measured in three different scenarios: before running the marketplace or idle state, running the marketplace in a normal execution, and executing the benchmarks mentioned above.

Finally, the power consumption of the RPi3 is measured in the same scenarios mentioned above, to visualize if the device reaches its peak consumption.

**Figure 11:**
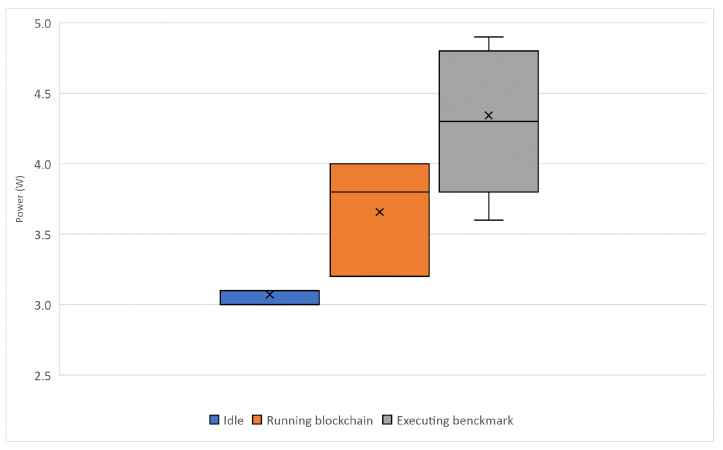
RPi3 power consumption in different scenarios.

As shown in Figure 11, RPi3 power consumption is near the 5W peak consumption when it is used to execute the benchmarks. However, it remains in a safe range of power consumption when running the marketplace, which has an impact on its durability and reliability over time.

## 8. Conclusions and Future Work

This paper has outlined a power marketplace, where blockchain and IoT are integrated to take advantage of its features, registering power offers and power demands from any actor in the system without third-party devices. Thus, this marketplace is suitable for the power industry, where different actors are involved in the smart grid.

Integration of blockchain and IoT in the same device can be possible for many different scenarios, one of which has been demonstrated in this paper. A marketplace to manage the power surplus could have actors in the network that are suited for most of the advantages that blockchain technology provides. Moreover, its implementation is made for low-performance, general-purpose devices by adapting the blockchain to its specifications. We have demonstrated that this can be achieved in a real network with a lightweight genesis block and a PoA consensus protocol, while still having enough performance to deploy some SCs in the network, at the same time as acting as an IoT node, with no performance problems in the devices.

When evaluating some key metrics on the nodes, the proposed marketplace results are light in terms of disk usage or CPU load. Therefore, it is a blockchain network that fulfills the purpose for which it was designed. This corroborates that this paper shows a valid integration of a blockchain in IoT devices, where the same device could have obtained data from some sensors and, in a different time window, register it to a blockchain network.

The following research on this topic refers to the optimization of IoT and blockchain integration. One of them is related to minimizing communications between nodes, as it is known that the SCs are triggered at specific points in time, so it would be desirable to send information between nodes only in case these data have been recorded in a block. In the same line, optimizing the consensus protocol, by changing or forking it, would be appropriate to reduce even more CPU load and RAM allocation.

This paper aims to be the start of a research line about optimizing IoT and blockchain integration in the same devices using GPU. As Geth, by how it is programmed, only uses the CPU for blockchain execution, the GPU is free for other purposes. Using both GPU and CPU at the same time, it would be possible to execute IoT algorithms at the same time. Furthermore, it would be possible to create a Geth fork that executes the blockchain code, taking advantage of the GPU, which would allow us to use other kinds of consensus algorithms in these devices. Other research lines would concern the specification of the tokenization of the energy market, in case the marketplace was a connection with real marketplaces, allowing trading among tokens.

## Figures and Tables

**Table 1:** Specifications of the selected devices.

	RPi3	RPi4 [54]	JNano [54]
CPU	Quad-core ARM Cortex-A53 @ 1.4 GHz	Quad-core ARM Cortex-A72 @ 1.5 GHz	Quad-core ARM Cortex-A57 @ 1.42 GHz
Memory	1 GB LPDDR2	8 GB LPDDR4	4 GB LPDDR4
Power mean Power peak	2.1 W 5 W	2.56 W 7.30 W	5 W 10 W

**Table 2:** Metrics of the implemented blockchain by node.

Metric	RPi4	RPi3	JNano	Units
Disk_ReadData	9745.44	1604.88	2058.76	bytes per hour
Disk_WriteData	11,495.96	2534.52	353.64	bytes per hour
Network_InboundTraffic	1573.60	68.13	68.17	bytes per hour
Network_OutboundTraffic	2123.52	68.24	68.44	bytes per hour
Memory_Allocated	3290.40	316.84	241.67	bytes per hour
Memory_AllocatedTotal	4.11 $\times \;10^{-5}$	3.17 $\times \;10^{-5}$	6.04 $\times \;10^{-6}$	%
CPU_ProcessesTime	1.55	3.58	2.03	%

**Table 3:** Benchmarks on RPi3: send rate and latencies in seconds (s).

Name	Send Rate (TPS)	Max Latency (s)	Min Latency (s)	Avg Latency (s)
25 TPS	25.0	7.43	2.09	4.72
50 TPS	50.1	7.71	2.15	4.90
100 TPS	99.0	9.44	2.71	6.50

**Table 4:** Benchmarks over RPi3: throughput, CPU usage peak, and CPU usage average.

Name	Throughput (TPS)	CPU% (Peak)	CPU% (Avg)
25 TPS	21.8	29.33	21.03
50 TPS	41.1	50.33	23.84
100 TPS	73.3	100	42.6

## Data Availability

Not applicable.
